# More Haste, Less Speed: Could Public–Private Partnerships Advance Cellular Immunotherapies?

**DOI:** 10.3389/fmed.2017.00134

**Published:** 2017-08-16

**Authors:** Tania Bubela, Katherine Bonter, Silvy Lachance, Jean-Sébastien Delisle, E. Richard Gold

**Affiliations:** ^1^Faculty of Health Sciences, Simon Fraser University, Burnaby, BC, Canada; ^2^Genome Canada Personalized Cancer Immunotherapy Program, Montreal, QC, Canada; ^3^Hematology-Oncology Division, Hôpital Maisonneuve-Rosemont, Montreal, QC, Canada; ^4^Department of Medicine, Université de Montréal, Montreal, QC, Canada; ^5^Faculty of Law, McGill University, Montreal, QC, Canada

**Keywords:** cellular immunotherapy, cancer, adoptive cellular transfer, CAR-T cell, public–private partnerships, Collaborative Research and Development Agreements, technology licensing

## Abstract

Cellular immunotherapies promise to transform cancer care. However, they must overcome serious challenges, including: (1) the need to identify and characterize novel cancer antigens to expand the range of therapeutic targets; (2) the need to develop strategies to minimize serious adverse events, such as cytokine release syndrome and treatment-related toxicities; and (3) the need to develop efficient production/manufacturing processes to reduce costs. Here, we discuss whether these challenges might better be addressed through forms of public–private research collaborations, including public–private partnerships (PPPs), or whether these challenges are best addressed by way of standard market transactions. We reviewed 14 public–private relationships and 25 underlying agreements for the clinical development of cancer cellular immunotherapies in the US. Most were based on bilateral research agreements and pure market transactions in the form of service contracts and technology licenses, which is representative of the commercialization focus of the field. We make the strategic case that multiparty PPPs may better advance cancer antigen discovery and characterization and improved cell processing/manufacturing and related activities. In the rush toward the competitive end of the translational continuum for cancer cellular immunotherapy and the attendant focus on commercialization, many gaps have appeared in our understanding of cellular biology, immunology, and bioengineering. We conclude that the model of bilateral agreements between leading research institutions and the private sector may be inadequate to efficiently harness the interdisciplinary skills and knowledge of the public and private sectors to bring these promising therapies to the clinic for the benefit of cancer patients.

## Introduction

Public–private partnerships (PPPs) are collaborative efforts to achieve mutually agreed objectives (1). They draw on the respective strengths and resources of the parties involved. In therapeutic product development, PPPs are based on complementary skills, materials, and knowledge along a translational continuum of research and development (R&D) by public/non-profit sector researchers and those in the biotechnology and/or pharmaceutical sectors. While considerable attention has been paid to PPPs engaged in the development of drugs and diagnostics or other devices, this perspective considers the role that PPPs might play in overcoming the clinical development and implementation challenges for cancer cellular immunotherapies. It first identifies the challenges, then takes a case-based approach to review public–private collaborative relationships in the US, and finally expands on the potential for multiparty PPPs to advance this promising field for the benefit of cancer patients.

## Clinical Development and Implementation Challenges for Cancer Cellular Immunotherapies

Cellular immunotherapies have been hailed as transformational for cancer care. In 2013, *Science Magazine* declared immunotherapy (cellular and checkpoint inhibitors) as its breakthrough of the year (2), and financial markets have generally concurred—2015 was a record year for investment in life sciences companies, with the greatest investment (1,496.49 Mill USD) in the category of immunotherapy/vaccines (3). The excitement stems, in part, from advances in adoptive cellular transfer (ACT), which uses chimeric antigen receptor (CAR-T) cells, tumor-infiltrating lymphocytes (TILs), or T cell receptor (TCR) engineered cells to recognize and target cancer cells (4). ACT promises to improve on the 2.5-month overall gain in survival time reported for cancer drugs approved between 2002 and 2014 (5). For example, clinical trials of CAR-T cells have reported positive results in acute lymphoblastic leukemia (ALL) (6), acute, relapsed refractory chronic lymphocytic leukemia (7), refractory multiple myeloma (8), and pediatric relapsed and refractory B-cell acute lymphocytic leukemia (B-ALL) (9). Similarly, TILs have shown great promise for metastatic melanoma (10–12).

Cellular therapies, in general, and cellular immunotherapies, in particular, face multiple challenges in clinical development and implementation. With respect to clinical development, leading cellular immunotherapy researcher, Dr. Steven A. Rosenberg, has identified lack of suitable targets as a major obstacle for cellular immunotherapies (13). If cellular immunotherapies are to be effective for solid tumors and for hematological malignancies, they must target cancer cells without causing off-target toxicities (14–16). Such toxicities, especially if unpredictable, will be a serious limiting factor for the clinical adoption of cellular immunotherapies. The identification of such cancer-specific antigens is therefore paramount for the future development of the field because most normal tissues, if destroyed by the cellular immunotherapy, cannot be replaced. Clinical trials have reported deaths from cardiopulmonary and neurological toxicities (14–16). Furthermore, cellular immunotherapy for B-cell leukemias that target CD19 may destroy normal B-cells. This can be palliated with immunoglobulin replacement (17). Hematological stem cell transplantation, often performed after these therapies, can also restore normal levels of immune cell subsets. Both, however, are delivered with Intensive Care Unit support, thereby increasing the cost of the therapies.

Cellular immunotherapies must also overcome their potential for other serious adverse events, primarily cytokine release syndrome. There appears to be a correlation between the efficacy of the immunotherapy in destroying cancer cells, and its adverse side effects—high efficacy in killing cancer cells may lead to a cytokine storm, especially in patients with a high disease burden (18). Neurotoxicity poses an additional risk. For example, in November 2016 leading cellular immunotherapy biotechnology company, Juno Therapeutics (Seattle, WA, USA), announced that it is placing a voluntary hold on the Phase II clinical trial of its leading CAR-T cell product, JCAR015, following the death of two participants with relapsed or refractory B cell ALL (19, 20). This voluntary hold for acute irreversible cerebral edema followed a hold placed on the same trial in July 2016 by the US Food and Drug Administration (FDA) due the deaths of three participants also from cerebral edema (21). At the time, Juno Therapeutics blamed the deaths on the addition of the chemotherapy, fludarabine, to eliminate the patient’s existing T-cells, making way for the CAR-T cells. The FDA lifted the hold only 1 week later (22). Not unexpectedly, the new November 2016 (without fludarabine) hold has had a dramatic effect on Juno Therapeutics shares; its stock price plummeted by 44% before trading was halted, and the impact of the deaths has spilled over to negatively impact other CAR-T cell companies (19, 20).

Even if cellular immunotherapy toxicities can be overcome, clinical implementation will be limited by the expected high cost of the therapies ($150,000–$500,000 per dose) that is, in part, determined by the emerging service-based autologous business model for cellular immunotherapies (23). ACT therapies currently derive from the cancer patient’s own circulating lymphocytes. Such autologous therapies incur substantial logistical challenges for scale-up. The circulating lymphocytes must be extracted from the patient, genetically manipulated (CAR or TCR transgenic T cells) or selected for antitumor effect (TILs), expanded, and then reinfused into the patient (12). Current business models suggest processing will occur in a centralized current Good Manufacturing Practice (cGMP) facility, while extraction and infusion will occur at a cancer center. Leading cellular immunotherapy companies, such as Juno Therapeutics and Kite Pharma (Santa Monica, CA, USA), are investing in cGMP infrastructure. The global pharmaceutical giant, Novartis (Basel, Switzerland), initially signaled its intent in the field by opening a Cell and Gene Therapies Unit and purchasing a New Jersey cGMP facility that was originally developed for bankrupt cancer vaccine company, Dendreon (Seattle, WA, USA). However, in February 2016, it closed the Unit to focus on its non-cellular cancer immunotherapy pipeline (24). This shift of Novartis toward its traditional business model cancer therapies, such as checkpoint inhibitors, may indicate continued skepticism in a viable business model for cellular therapies (25). To the detriment of the field, autologous therapies have so far demonstrated greater efficacy than generic allogeneic products. Nevertheless, Cellectis (Paris, France) has advanced an allogeneic CAR-T immunotherapy derived from T cell precursors manipulated using TALEN^®^ technology into Phase I clinical trials (2015-004293-15). The product has been developed in collaboration with Pfizer and Servier and therefore does not represent a PPP. However, Cellectis has entered into a research and development alliance with researchers at MD Anderson Cancer Center (TX, USA), discussed below. The development of allogeneic cellular immunotherapies will be a fruitful area for future PPPs. Advances in all aspects of the service pipeline are therefore central to the clinical success of cellular immunotherapy.

## Analysis of Current Public–Private Relationships for Cancer Cellular Immunotherapy

Public–private partnerships are one form of research collaboration based on shared decision making by the public and private sector parties involved with respect to goals, membership, ongoing management, potential expansion of the collaboration, and distribution of benefits (26). Such partnerships harness the complementary skills of the parties along the translational continuum from research laboratory to clinical trials, recognizing that the pathway for most therapies is neither certain nor linear, especially for novel treatment paradigms such as cellular immunotherapy. Rather, the pathway involves iterative research and development as successive challenges in safety and efficacy are identified and sometimes addressed.

Many biomedical PPPs are supportive of the precompetitive portion of the translational continuum wherein they facilitate the sharing of tacit knowledge (27), data, and materials, without limiting the ability of specific actors to appropriate knowledge that is closer to practical application (28–30). As such, PPPs stand in contrast to pure market transactions based on service contracts and technology licensing that more clearly delineate the rights and responsibilities of parties in a competitive environment (26). For example, a research-intensive, precompetitive PPP may be based on a consortium agreement between multiple members that sets out a shared governance structure. In contrast, relationships based on market transactions rarely establish the shared governance models that characterize PPPs. An intermediate form is a hub and spoke model whereby a central party enters into bilateral research agreements with multiple parties to advance its centralized goal. The ordering of research relationships from shared governance structures and collaborative research agreements to service contracts and technology licenses mirrors the translational continuum, from precompetitive to competitive research. The constellation of agreements will depend on the maturity of the technology in question and the state of certainty about its efficacy and market.

The preceding section identified four challenges that might be better addressed by PPPs, given the nascent stage of the field of cellular immunotherapy: (1) the need to identify novel cancer antigens to expand the range of therapeutic targets and minimize both off-target effects and on-target but off-cancer effects; (2) the need to develop strategies to minimize serious adverse events, such as cytokine release syndrome; (3) the need to develop allogeneic therapies; and (4) the need to develop efficient production/manufacturing processes to reduce costs. The issue is whether these challenges might be better addressed through forms of public–private research collaborations, including PPPs, or whether these challenges are best addressed by way of standard market transactions. In this section, we review public–private research relationships in the US. In the next section, we discuss how PPPs might improve the clinical translation of cellular immunotherapies.

The focus of our review was on PPPs that had developed products in clinical trials up to December 2015. For our review, we selected 14 US public–private relationships for the clinical development of cancer cellular immunotherapies based on a comprehensive analysis of 1,579 interventional clinical trials from global registries, of which 329 were industry sponsored (31). Of these, 35 companies had products in clinical development beyond Phase I, with verified status as of September 2016. Of these 35 companies, 34 were biotechnology companies operating in Western Europe (*n* = 16) and North America (*n* = 17), and one was the pharmaceutical company, Novartis. We reviewed the history of the public–private relationships of Novartis and the 11 North American companies whose clinical trial registry entry indicated that their product was still in clinical development (i.e., not terminated or withdrawn) and listed at least one collaboration with a research institute (Table 1). This is a limitation of our review—we only identified collaborations from the clinical trial record; we did not contact companies or interview investigators associated with all the industry-sponsored clinical trials and may therefore have missed some collaborations with academic centers.

**Table 1:** Public–private collaborative efforts in the US of cancer cellular immunotherapy in Phase II and III clinical trials.

Company sponsor	Collaborators	Public/private	IPO year/founding year	Product	Cell type^a^	Cell source	Target^b^	Condition	Clinical trial phase in September 2016 and identifiers
Argos Therapeutics	Rockefeller University; Duke University	Public: NASDAQ: ARGS	2014	Rocapuldencel-T	DC	Auto	TERT, OFA, G250 + CD40L	Renal cell carcinoma	3 (started 2012, active) NCT01582672
Asterias Biotherapeutics	Cancer Research UK; Cell Therapy Catapult	Public: NASDAQ: AST	2016	GRNVAC1	DC	Auto	hTERT	Acute myeloid leukemia	2 (completed 2011) NCT00510133
Atara Biotherapeutics	Memorial Sloan Kettering; Amgen; Celgene	Public: NASDAQ: ATRA	2014	EBV-CTL	T	Allo	EBV	Non-Hodgkin’s lymphoma	2 (started 2011, still recruiting) NCT01498484
Cell Medica	Baylor College of Medicine; University College London	Private	2006	CMD-003	T	Auto	EBV	Non-Hodgkin’s lymphoma	2 (started 2014, recruiting) NCT01948180
ImmunoCellular Therapeutics	Cedars-Sinai Medical Center	Public: NASDAQ: IMUC	2006	ICT-107	DC	Auto	AIM-2, MAGE-1, TRP-2, gp100, HER-2, IL-13Ra2	Glioblastoma	3 (started 2015, recruiting) NCT02546102
Juno Therapeutics	Fred Hutchinson Cancer Research Center; St. Jude Children’s Research Hospital; Memorial Sloan Kettering Cancer Center; Seattle Children’s Research Institute	Public: NASDAQ: JUNO	2014	JCAR015	CAR-T	Auto	CD19	Acute lymphoblastic leukemia (ALL)	2 (started 2015, recruiting) NCT02535364
Kite Pharma	National Cancer Institute; UCLA David Geffen School of Medicine; Tel-Aviv Sourasky Medical Center; Leiden University Medical Center; Alpine Immune Science	Public: NASDAQ: KITE	2014	KTE-C19	CAR-T	Auto	CD19	Mantle cell lymphoma	2 (started 2015, recruiting) NCT02601313
Lion Biotechnologies	National Cancer Institute	Public: NASDAQ: LBIO	2010	Contego (LN-144)	TIL	Auto	TS	Melanoma	2 (started 2015, recruiting) NCT02360579
Northwest Biotherapeutics	King’s College London	Public: NASDAQ: NWBO	2001	DCVax-L	DC	Auto	TS	Glioblastoma	3 (started 2006, ongoing) NCT00045968
TVAX Biomedical	National Cancer Institute; University of Kansas Medical Center	Private Spin off from University of Kansas	2004	TVI-Brain-01	CTL	Auto	TS	Grade IV glioma	2 (started 2011, recruitment status not verified) NCT01290692
Novartis Pharmaceuticals (Switzerland)	National Cancer Institute, University of Pennsylvania	Public: VTX: NOVN	1996	Tisagenlecleucel-T (CTL019, CART19)	CAR-T	Auto	CD19	ALL	2 (started 2015, recruiting) 2 (started 2014, recruiting) NCT02445248 NCT02435849 NCT02228096

*^a^Cell type: (+), multiple agents per product; DC, dendritic cell; CTL, cytotoxic T-cells (CD8+); CAR-T, chimeric antigen receptor T cell; T, T cell; TIL, tumor-infiltrating lymphocyte*.

*^b^Target: EBV, Epstein–Barr virus; TS, patient tumor sample; TCL, tumor cell line; CTA, cancer testis antigens*.

We identified 23 separate agreements. In addition, our review of the academic literature and biotechnology news coverage by STAT News and FierceBiotech of cancer cellular immunotherapy identified four additional agreements by US companies of interest, whose product or technology development fills a gap to an identified challenge: Bellicum Pharmaceuticals (Houston, TX, USA), bluebirdbio (Cambridge, MA, USA), Cellectis (Paris, France), and Adaptimmune Therapeutics (Abington, UK). We further reviewed the history and nature of the research relationships based on documents identified in biotechnology and pharmaceutical trade publications (Factiva database), company websites and SEC filings, and contracts—10 of which had a full-text version available on the Recap database (confidential details redacted).

Our review of the 25 agreements identified a mixture of collaborative research agreements and pure market transactions in the form of service contracts and technology licensing (Table 2). The research agreements for collaborations between companies and research institutions (including universities and hospitals) were based on a hub and spoke model. In other words, when a company listed multiple collaborators on its sponsored clinical trial, all of the research agreements were bilateral between the company and the research institute. Our search only identified two relationships that clearly articulated a shared governance structure (likely an underestimate based on publicly available data we accessed rather than interviews with the parties). Cell Medica’s (London, UK) separate agreements with Baylor College of Medicine and the University College London both stated that the research would be conducted under the guidance of a Joint Steering Committee, with representatives from each party to the respective agreement. In the agreement with University College London, either party could bring novel targets or platform technologies to the collaboration. Note that these agreements specifically stated their stage of research as preclinical and early clinical, after which, Cell Medica had an exclusive license and option agreement to move forward with the codeveloped technologies. Indeed, the majority of the collaborative research agreements we identified additionally provided for options or licenses to the technologies developed.

**Table 2:** Nature of the relationship between companies and research institutions in the development of cancer immunotherapies.

Company sponsor^a^	Collaborators	Collaborative research relationship^a^	Technology licensing/service agreements^a^
Argos Therapeutics	Rockefeller University and Duke University		Cofounders of company were researchers the two universities who discovered role of dendritic cells in the immune system and developed a method to generate dendritic cells (Rockefeller) and developed a unique RNA-based dendritic cell technology (Duke)
Asterias Biotherapeutics	Cancer Research UK; Cell Therapy Catapult	2015: The collaboration with the Cell Therapy Catapult will trigger the initiation of an Asterias subsidiary in the UK to more effectively collaborate with Cancer Research UK and the Cell Therapy Catapult^a^	2014: Service agreement between Cancer Research Technology and Asterias for product manufacturing of cancer biotherapeutics 2015: Service contract to develop scaled production procedures with Cell Therapy Catapult. The program will utilize the know-how and resources assembled at the Cell Therapy Catapult along with expertise in pluripotent stem cells at Asterias to industrialize production of pluripotent stem cell-based therapeutics^a^
Atara Biotherapeutics	Memorial Sloan Kettering (MSK); Amgen; Celgene	2014: Parties agreed to collaborate on further research to develop additional cellular therapies, including against other antigens or CAR-T cells	2014: Worldwide exclusive option agreement from MSK for the development and commercialization of T-cells activated against: EBV, CMV, and WT1 in exchange for cash and Atara common. If Atara exercises its option, MSK will receive an upfront license payment and be eligible to receive additional payments based on achievement of development, regulatory and sales-related milestones, as well as royalty payments 2015: Atara exercised its exclusive option for the three programs to expand its pipeline after EBV target received Food and Drug Administration breakthrough-therapy designation
Adaptimmune Therapeutics	MD Anderson Cancer Center	2016: Announced a multiyear strategic alliance to expedite the development of novel adoptive T-cell therapies for multiple types of cancer, targeting	2016: The alliance pairs preclinical and clinical teams from the MD Anderson with Adaptimmune Therapeutics’ Specific Peptide Enhanced Affinity Receptor (SPEAR^®^) T-cell technology platform that enables the identification of targets (e.g., MAGE-A10 and MAGE-A4) expressed on solid and hematological cancers and to develop affinity-enhanced TCRs with optimal potency and specificity against them
Cellectis	MD Anderson Cancer Center	2015: Cellectis and MD Anderson have entered into a research and development alliance that aims to develop novel allogeneic cellular immunotherapies	2015: The Alliance aims to build on MD Anderson’s preclinical and clinical expertise in leukemia and myeloma coupled with Cellectis’ first-in-class allogeneic CAR T-cell therapeutic approach and manufacturing capabilities
Cell Medica	Baylor College of Medicine (Dr. Leonid Metelitsa)	2016: Codevelopment partnership with Baylor College of Medicine (Baylor) to develop next-generation technologies (CAR, NKT, and TCR) for engineering immune cells with enhanced functions for the treatment of solid tumors. Within the codevelopment structure, Baylor will conduct the preclinical and Phase I clinical research under the guidance of the Joint Steering Committee. Cell Medica will work in parallel to support early product development and will use its substantial experience in manufacturing clinical-grade cell therapies to establish robust production processes suitable for industrial scale-up	2016: License and Option Agreement for two platform patents related to engineered NKT cells, three target cancer antigens for CAR-modified NKT cells, and a TCR technology. Cell Medica has paid an upfront fee for the exclusive licensing arrangements and will make additional payments to exercise its exclusive option to license future products
Cell Medica	University College London (Profs. Hans Stauss and Emma Morris)	2016: Research collaboration to utilize UCL’s novel TCR technology to generate TCR products for cancer treatment. UCL will conduct the preclinical and early clinical research under the guidance of a Joint Steering Committee. As part of this agreement, both parties can bring targets or platform technologies to the collaboration, aiming to generate leading edge modified TCR products. Cell Medica will support product development with expertise in manufacturing clinical-grade cell therapies and establishing robust production processes suitable for industrial scale-up	2016: Exclusive license and option agreement with UCL Business for TCR platform patent and two target antigens. Cell Medica has paid an upfront fee and will make additional payments to exercise its exclusive option to license future products. UCL is eligible to receive further payments related to clinical, regulatory and sales milestones, as well as single digit royalties
ImmunoCellular Therapeutics	Cedars-Sinai Medical Center, Los Angeles		2015: Company was founded following the acquisition of cellular-based technology from Cedar Sinai ImmunoCellular Therapeutics that was established in 2006 with cellular-based technology licensed from the Cedar-Sinai Medical Center. Technology included dendritic cell-based vaccines for brain tumors and other cancers and neurodegenerative disorders. In 2012, the company also exclusively licensed related technologies for specific cancers from the University of Pennsylvania
Juno Therapeutics	St. Jude Children’s Research Hospital		2013: Exclusive license for IP related to JCAR014 and JCAR017, genetically engineered autologous T lymphocytes for cancer. Royalty payments based on clinical and development milestones^a^
Juno Therapeutics	Seattle Children’s Research Institute		2013: Exclusive license for IP related to the development and commercialization of lead cancer immunotherapy CAR-T products: JCAR014 and JCAR017^a^
Juno Therapeutics	Fred Hutchinson Cancer Research Center		2013: Exclusive license for IP related to JCAR014 and JCAR017, genetically engineered autologous T lymphocytes for cancer^a^
Kite Pharma	National Cancer Institute (Dr. Steven A. Rosenberg)	2012: CRADA for the development and commercialization of novel engineered peripheral blood autologous T cell therapeutics for the treatment of multiple cancer indications 2015: Amended CRADA for expanded tumor neo-antigens and CAR-T products for solid tumors^a^	
Kite Pharma	National Cancer Institute (Dr. James N. Kochenderfer)	2012: CRADA for engineered peripheral blood autologous T cell therapeutics (eACT) for hematological and solid cancers^a^ 2013/5: Research collaboration for engineered peripheral blood autologous T cell therapeutics (eACT) for hematological and solid cancers^a^ 2012/5: Research collaboration for engineered peripheral blood autologous T cell therapeutics (eACT) for hematological and solid cancers^a^ 2016: CRADA for fully human anti-CD19 CAR product for B-cell lymphomas and leukemias	2012/3 and 2012/5: Options for exclusive license for engineered peripheral blood autologous T cell therapeutics (eACT) for hematological and solid cancers^a^ 2014: Exclusive license for IP related to TCR-based products against HPV-16 E6 and E7 oncoproteins for cancers associated with HPV infection
Kite Pharma	National Cancer Institute	2014: CRADA for research and clinical development of TCR product candidates directed against HPV-16 E6 and E7 oncoproteins^a^	2014: Exclusive license for IP related to TCR-based products against HPV-16 E6 and E7 oncoproteins for cancers associated with HPV infection
Kite Pharma	University of California, Los Angeles (UCLA)		Exclusive, worldwide license agreement for technology to advance the development of off-the-shelf allogeneic T-cell therapies from renewable pluripotent stem cells
Kite Pharma	Tel-Aviv Sourasky Medical Centre (Prof. Zelig Eshhar: 2013 appointed to Scientific Advisory Board Kite Pharma)	2015: Research agreement for collaboration on peripheral autologous T-cell therapeutics on CAR or TCR platforms	
Kite Pharma	Leiden University Medical Centre (LUMC)	2016: Research agreement to identify and develop TCR product candidates targeting solid tumors associated with the HPV type 16 infection	Option to license multiple TCR gene sequences for the development and commercialization of product candidates
Genesis Biopharma (GB) founded in 2007 with SAB member Rosenberg merged with Lion Biotechnologies in 2013^a^ (Dr. Steven A. Rosenberg)	National Cancer Institute	2011: CRADA with GB to develop TILs designed to destroy metastatic melanoma cells using a patient’s tumor infiltrating lymphocytes^a^ 2015: LB amended CRADA to include 4 new tumor indications for TIL therapy^a^ 2016: extended CRADA for another 5-year term to 2021. Includes development of TIL therapy for treatment of metastatic melanoma, bladder, lung, breast, and HPV-associated cancers^a^	
Northwest Biotherapeutics (NW Bio)	Kings College, London Cognate BioServices Inc. is NW Bio’s contract manufacturer for DCVax^®^	2001: Manufacturing and clinical trials partnership whereby trials for DCVAx for GBM conducted at King’s College Hospital with expanded access program, and Cognate BioServices, Inc. provides technology transfer and training in proprietary DCVax production processes, adding manufacturing capacity and flexibility without need for further investment by NW Bio	
NW Bio	Fraunhofer Institute for Cell Therapy and Immunology	In addition to same terms as above. The partnership makes NW Bio eligible for certain grants through the German government, which, if approved, could amount to as much as 2–3 million Euro	
TVAX Biomedical	National Cancer Institute; University of Kansas Medical Center		Start-up company from the University of Kansas Medical Center formed to commercialize TVAX immunotherapy for personalized cancer treatment
Novartis Pharmaceuticals (Switzerland)	National Cancer Institute, University of Pennsylvania	2012: 5-year global collaboration to research, develop and commercialize targeted CAR immunotherapies for cancer treatment and to build a first-of-its-kind Center for Advanced Cellular Therapies on the Penn campus in Philadelphia at a cost of $20 Mill USD	2012: Penn grants Novartis an exclusive worldwide license to the technologies used in an ongoing trial of patients with CLL with CTL019 as well as future CAR-based therapies developed through the collaborations. Milestone and royalty payments to Penn
Bellicum Pharmaceuticals	LUMC		2015: Research agreement under which Bellicum will provide LUMC with funding for research to discover and validate high-affinity TCR product candidates targeting several cancer-associated antigens. Bellicum receives option to obtain an exclusive, worldwide license to practice and exploit the inventions
Bluebirdbio and Celgene	Center for Cell and Gene Therapy at Baylor College of Medicine, Texas Children’s Hospital and The Methodist Hospital, Houston (Dr. Malcolm Brenner)	2013: bluebirdbio, Celgene, and Dr. Brenner will work collaboratively to advance and develop existing and new products and programs in the CAR T-cell field. Financial terms include upfront payment and up to $225 Mill USD per product in potential option fees and clinical and regulatory milestones	bluebird bio has the right to participate in the development and commercialization of any licensed products resulting from the collaboration through a 50/50 codevelopment and profit share in the US in exchange for a reduction of milestones

*CAR, chimeric antigen receptor; CLL, chronic lymphocytic leukemia; CRADA, Collaborative Research and Development Agreement; DC, dendritic cell; EBV, Epstein–Barr virus; GBM, glioblastoma multiforme (brain cancer); HPV, human papillomavirus; IP, intellectual property; NKT, natural killer T cells; TCR, T cell receptor; TIL, tumor infiltrating lymphocyte; WT1, Wilms tumor 1*.

*^a^Contract(s) available for review from Recap database*.

One benefit of PPPs may be in the efficient transfer of tacit knowledge (know-how) through collaborative interactions. However, our review demonstrated that market transactions in cellular immunotherapy may also account for such knowledge transfer (Table 2). For example, the agreement between Asterias Biotherapeutics (Fremont, CA, USA) and Cancer Research UK provided for negotiations to adapt the technology transfer plan associated with a joint development project to use the company’s expertise in cell-manufacturing and industrial scale-up to improve manufacturing/production of the research institute’s cellular immunotherapy candidates. The company committed to the transfer of manufacturing/production know how, including in the form of training of research institute staff. Similarly, the agreement between Northwest Biotherapeutics (NW Bio) (Bethesda, MD, USA) and Kings College London provided for technology transfer and training *via* its manufacturing service provider, Cognate BioServices (Memphis, TN, USA).

Another stated benefit of the collaborative agreements (especially with UK/European research institutes) was access to public funding, for example access to German funding in the agreements between the Fraunhofer Institute for Cell Therapy and Immunology and NW Bio, and between Asterias Biotherapeutics and Cancer Research UK.

In the US, the preferred model for research agreements between the National Institutes of Health (NIH) and companies is the Collaborative Research and Development Agreement (CRADA). CRADAs provide the legal framework for investigators from these two sectors to conduct research in pursuit of common goals, while leveraging their own research resources: “The purpose of a CRADA is to make Government facilities, intellectual property, and expertise available for collaborative interactions to further the development of scientific and technological knowledge into useful, marketable products” (32). All collaborators must make significant intellectual contributions to the research project or contribute materials and resources not available at the NIH. CRADAs are distinct from sponsored research. CRADAs are not a general funding mechanism, but are specific in their support of the collaborative project. Their terms ensure research freedom and may not unreasonably restrict or constrain the dissemination of research information. Nevertheless, they do support the protection of proprietary materials and intellectual property rights and may grant the industry partner an option to exclusively license intellectual property.

The seven CRADAs we identified also were bilateral in form (Table 2). However, they notably covered the identification of new cancer antigens for targeted cellular immunotherapy. This result is representative of a traditional role of research institutes in target identification for drug discovery. Target identification may be based on review of the peer-reviewed literature followed by early-phase trials to demonstrate safety and proof-of-concept in humans (33). Since most targets will prove neither safe nor efficacious, the public sector plays an important role in de-risking these for later stage development, including through research to enhance understanding of the molecular biology and possible mechanisms of action. Indeed, eight of the research agreements and six of the license agreements we identified explicitly mentioned new antigens/products as a goal.

Finally, we added Bellicum Pharmaceuticals and bluebirdbio to our list of companies because they are explicitly developing technologies that derive from university-based research to mitigate adverse events. These companies are developing molecular switch technologies for programmed cell death of CAR-T or similar cells or to mute CAR-T cell therapy associated adverse events, respectively. We also added Adaptimmune Therapeutics because it is an example of a strategic alliance for target identification. The company has entered into a strategic alliance that combines the companies T-cell technology platform that enables the identification of targets expressed in solid and hematological cancers with MD Anderson Cancer Center’s expertise in preclinical and clinical research (Table 2). Finally, we added Cellectis, which has entered into a research and development alliance, also with MD Anderson Cancer Center, to use the company’s CAR-T cell therapeutic approach and manufacturing technology and MD Anderson Cancer Center’s research expertise to develop allogeneic CAR-T cells. The latter technology has the potential to simplify the business models for manufacture and delivery of CAR-T therapies, thereby reducing costs.

## Could PPPs Advance Cancer Cellular Immunotherapy?

Our review focused on collaborations for clinical development beyond Phase I, which may, in part explain, why we found limited evidence that the products in development resulted from precompetitive PPPs. The public–private relationships we identified were based on bilateral collaborative agreements between companies and research institutions for research based on a common goal. They rarely identified shared governance mechanisms, but rather relied on a hub and spoke model for research relationships. This focus on bilateral agreements and pure market transactions in the form of service contracts and technology licenses is representative of the commercialization focus of the field. The rapid advancement of cellular therapeutics comes with attendant hype with respect to potential efficacy and market size, as evidenced by media and other coverage and a rapid increase in the number of clinical trials and investment in private-sector companies (34).

The market enthusiasm for cellular therapeutics exists in spite of serious concerns about adverse events, business models, and the complexity of cells as therapies (34, 35). Indeed, the latest deaths in Juno Therapeutics’ clinical trial have brought criticism that therapies are being tested in terminal cancer patients without adequate understanding of their biological mechanisms and potential for adverse events (19, 20). This lack of mechanistic understanding presages an expanded role for pre- and early-stage clinical research in the province of research institutions. It may be summed up by the saying “more haste, less speed,” which is defined by the Cambridge English Dictionary as meaning that if you try to do things too quickly, it will take you longer in the end.

In addition to an enhanced role for academic-industry collaborations in overcoming adverse events, we identified one case—Asterias Biotherapeutics—that exemplified the role of PPPs in the development of production/manufacturing (Table 2). This implies a greater role for not only the clinical research community, but also for bioengineers that specialize in cell processing, manipulation, sorting, and expansion to clinical dosage levels (25, 36). The research agreements we identified were focused on clinical partnerships and therefore raise opportunities for an expanded set of interdisciplinary partners. At this juncture, there is an important role for industry expertise, as evidenced by some agreements for cGMP scale-up for clinical application.

Finally, there is a clear convergence of interests between research institutions and industry in the identification and preclinical characterization of novel cancer antigens, both to expand the types of tumors that may be targeted by cellular immunotherapies and reduce on-target, off-cancer adverse effects. As stated above, the de-risking of novel targets falls within the purview of research institutions and target identification has been the subject of successful PPPs. The best known of these is the Structural Genomics Consortium (SGC) that creates an open collaborative network of scientists across sectors to identify druggable protein targets and develop chemical probes for drug discovery (29, 37). The differences between the SGC and the research collaborations we identified are the large number of partners within the SGC and its commitment to open science (38). Its open science model and common governance structure stands in contrast to a proprietary model based on options to license codeveloped intellectual property. PPPs such as the SGC bring the added benefit of enabling systematic, high-throughput research that avoids duplication of effort and reduces costs.

While the SGC is built on an open science model, other PPPs enable commercialization based on formal intellectual property rights within an open innovation platform. Such a model may be more palatable in the context of cellular immunotherapy, given the rapid advance to clinical translation in the field and the fact that the field is dominated by biotechnology rather than larger pharmaceutical companies. One example is the European Lead Factory, a pan-European drug discovery project of 30 partners established in 2013, which has received E196 million in funding from the Innovative Medicines Initiative and other sources (39). The European Lead Factory supports the generation of a compound library and an industry-standard screening center, providing free access to around 500,000 novel compounds. Any researcher from a European academic center or a small- and medium-sized enterprise (SME) can apply to screen a drug target of interest and to which the researcher/SME has intellectual property rights. If a screening application is accepted by the European Lead Factory, the parties enter into a standard contract that ensures confidentiality of the screening program and resulting data. Researchers/SMEs receiving the results are able to manage them as they see fit, but are given the option to partner with one of the participating pharmaceutical companies. Researchers are free to make results public, following the PPP’s publication guidelines. However, if the screening program results in patent rights, there is an obligation to share benefits with the European Lead Factory. The researcher/SME can pay the PPP a fixed amount while filing the patent, a higher amount 2 years following filing, or a percentage of royalties generated by the patent.

Given that cellular immunotherapies are highly personalized, autologous therapies, it is expected that there might be an additional convergence in the discovery of cancer targets for cellular immunotherapies and precision medicine initiatives. The latter are building PPPs focused on the identification and development clinical protein-based biomarkers. For example, the Personalized Medicine Partnership for Cancer is a public–private consortium, in part funded by the Government of Québec, Canada (http://pmpc-org.com/en/). It partners a Quebec-based multidisciplinary network of clinicians, academic scientists and other members of the translational research community with private-sector partners: Caprion, a Montreal-based biotechnology company, Oncozyme Pharma (Montreal, QC, Canada), Pfizer Canada (Kirkland, QC, Canada), and Sanofi Canada (Laval, QC, Canada). Exemplifying the convergence between biomarker and cancer antigen discovery, in 2016, Caprion presented results on the use of its platform to identify neo-epitopes for cancer vaccines and adoptive T-cell therapies (40). Similarly, in 2012, the German Ministry for Education and Research granted 1.2 Mill Euro over 3 years to a public–private Consortium of Individualized Vaccines for Cancer (41).

In conclusion, a strategic case may be made to establish multiparty PPPs with governance structures to advance two areas that are crucial to the safe and effective translation of cellular immunotherapies for cancer: cancer antigen discovery and characterization and improved cell processing/manufacturing and related activities. This conclusion is supported in the Recommendations of the Blue Ribbon Panel on *Research Opportunities for the Vice President’s Cancer “Moonshot,”* which may still proceed in some form under the new US administration, identified a strategic need for better coordination for data and tumor samples from cancer patients that may benefit from a series of PPPs (42). To advance immunotherapies, it recommended the integration of methods and sequencing data, especially with respect to proteins that are uniquely expressed in pediatric cancers, supported by the integration of PPPs “to develop the right immunotherapeutic tools (drugs) to exploit these targets” (42).

In the rush toward the competitive end of the translational continuum for cancer cellular immunotherapy and the attendant focus on commercialization of research, many gaps have appeared in our understanding of cellular biology, immunology, and bioengineering. In the US, the model of bilateral agreements between leading research institutions and the private sector may be inadequate to efficiently harness the interdisciplinary skills and knowledge of the public and private sectors to bring these promising therapies to the clinic for the benefit of cancer patients.

## Author Contributions

TB designed the review, drafted, edited, and submitted manuscript; KB, SL, J-SD, and EG contributed to the review, commented on draft manuscript, and approved submission.

## Conflict of Interest Statement

The authors declare that the research was conducted in the absence of any commercial or financial relationships that could be construed as a potential conflict of interest.
